# Salt Stress Amelioration in Maize Plants through Phosphogypsum Application and Bacterial Inoculation

**DOI:** 10.3390/plants10102024

**Published:** 2021-09-27

**Authors:** Tamer Khalifa, Mohssen Elbagory, Alaa El-Dein Omara

**Affiliations:** 1 Agricultural Research Center, Department of Soil Improvement and Conservation, Soils, Water and Environment Research Institute, Giza 12112, Egypt; tamerkhalifa1985@gmail.com; 2 Department of Biology, Faculty of Science and Arts, King Khalid University, Mohail Assir 61321, Saudi Arabia; mhmohammad@kku.edu.sa; 3 Agricultural Research Center, Department of Microbiology, Soils, Water and Environment Research Institute, Giza 12112, Egypt

**Keywords:** phosphogypsum, PGPR, soil salinity, nutrient content, maize productivity

## Abstract

The use of phosphogypsum (PG) and plant growth-promoting rhizobacteria (PGPR) for agricultural purposes are good options to improve soil properties and increase crop yield. The objective of this study was to investigate the effect of different rates of PG (ton ha^−1^; 0 (PG1), 3 (PG2), 6 (PG3), and 9 (PG4)) combined with PGPR inoculation (*Azospirillum lipoferum* (control, T1), *A. lipoferum* + *Bacillus coagulans* (T2), *A. lipoferum* + *B. circulance* (T3), and *A. lipoferum* + *B. subtilis* (T4)) on soil properties, plant physiology, antioxidant enzymes, nutrient uptake, and yield of maize plants (*Zea mays* L., cv. HSC 10) grown in salt-affected soil. Over two growing seasons, 2019 and 2020, field experiments were conducted as a split-plot design with triplicates. The results show that applying PG (9 ton ha^−1^) and co-inoculation (*A. lipoferum* + *B. circulance*) treatment significantly increased chlorophyll and carotenoids content, antioxidant enzymes, microbial communities, soil enzymes activity, and nutrient contents, and showed inhibitory impacts on proline content and pH, as well as EC and ESP, thus improving the productivity of maize plant compared to the control treatment. It could be concluded that PG, along with microbial inoculation, may be an important approach for ameliorating the negative impacts of salinity on maize plants.

## 1. Introduction

In arid and semi-arid regions, soil salinity has been reported to have a negative impact on soil quality and crop growth, affecting 25 to 30% of the crop productivity of agricultural soils [1,2,3]. In addition, salinity is a major environmental stress and a major obstacle to crop production. The salinization of arable land is expected to have devastating effects globally, resulting in 30% of lost land over the next 25 years and up to 50% by the mid-21st century [4]. Nowadays, 50% of the world’s total cultivated area is exposed to salinity stress, causing a loss of about USD 12 billion [5]. In addition, soil salinity is a complex process that has negative effects on the activity of physiological and biochemical processes in the plant [6]. Therefore, the toxicity of specific ions during metabolic processes or osmotic stress leads to a reduction in plant growth, nutrient uptake, and enzyme activity [7,8]. On the other hand, the adverse effects of salt-affected soils are associated with the reduced osmosis (primary stage) and cytotoxicity of ions (secondary stage), as well as the production of reactive oxygen species (ROS) and nutrient imbalance [9]; high osmotic stress is related to the accumulation of soluble salts in the soil solution, which leads to water stress [10]. According to [11], the inhibitory effect of salinity stress on the growth and productivity of different crops depends on several factors, such as salt concentration in the soil solution, time interval, plant species, plant growth stage, gas exchange properties, photosynthetic pigments, and environmental conditions. Therefore, it is important to explore sustainable management practices to reduce the harmful effect of this problem through genetic techniques, breeding programs of crops for salinity tolerance, soil conditioners, and biological products [12,13,14]. Within these practices, beneficial microorganisms (plant growth-promoting rhizobacteria, PGPR) play an important role in ameliorating salt stress and the ability to release nutrients from organic and inorganic forms to soluble forms, which appears to be a promising process for enhancing the tolerance of crops to salinity [15]. Several researchers have suggested that PGPR may be applicable in enhancing crop production under salt stress by enhancing the nutrient uptake physiological modifications, and antioxidant activities of plants [16,17,18]. Several PGPR genera, including *Pseudomonas*, *Bacillus*, *Azospirillum*, *Bradyrhizobium*, and *Flavobacterium* are known to improve the growth and productivity of different crops grown in salinity-affected soils. PGPR can be attached to the roots of plants, such as endophytes, rhizoplane, or rhizosphere [19,20]. 

Rock-bearing nutrients, along with plant growth-promoting bacteria, are a recent alternative strategy to synthetic chemical fertilizers. In this context, phosphogypsum (PG) is a by-product of the phosphate fertilizer industry, resulting from the production of phosphoric acid from rock phosphate (fluorapatite). Worldwide, about 160 million tons of phosphogypsum are produced annually and it is primarily disposed of in large stocks or discharged into water bodies [21]. It contains mostly sulfur, calcium oxide, and small amounts of phosphorous [22] and heavy metals, as well as impurities of radioactive elements [23]. As the hazardous effects of PG waste in the environment are increasing due to continued industrialization, proper management is required to minimize the adverse effects on humans and the ecosystem [24]. In different countries around the world, phosphogypsum is used in agriculture either as a soil amendment (calcium sulfate) or as fertilizer [25], which may be an efficient amendment to reclaim alkaline soils by improving the soil properties and the availability of different nutrients to crops (direct) or in combination with microbes to enhance the solubility of nutrients (indirect) [26]. Several investigators have suggested that synergistic application with PG and microbial inoculation increased the levels of NPK uptake and enhanced the growth of maize plants [26], enhancing the microbial biomass carbon, N concentrations, soil enzyme, and plant growth [27,28]. Therefore, the objective of this study was to investigate the application effects of different rates of PG combined with microbial inoculation on chemical and biological soil properties, plant physiology, antioxidant enzymes, the contents of macro- and micronutrients, and the yield of maize plants grown in salt-affected soil.

## 2. Results

### 2.1. Physiological Characteristics in Maize Leaves

In both seasons (2019 and 2020), the contents of the chlorophyll (mg g^−1^ FW), carotenoids (µg g^−1^ FW), and proline (µmole g^−1^ FW) of maize leaves at 60 days after sowing showed significant differences (*p* < 0.05) with respect to different applications of phosphogypsum (PG1 (0 t ha^−1^), PG2 (3 t ha^−1^), PG3 (6 t ha^−1^), and PG4 (9 t ha^−1^)) and microbial inoculations (*A. lipoferum* (control, T1), *A. lipoferum* + *B. coagulans* (T2), *A. lipoferum* + *B. circulance* (T3), and *A. lipoferum* + *B. subtilis* (T4)) (Table 1). Generally, highly significant differences were observed for the chlorophyll and carotenoids parameters with increasing PG rates and microbial inoculations, and thus, the results occurred in the descending order of PG4 > PG3 > PG2 > PG1 for different phosphogypsum rates and T3 > T2 > T4 > T1 for different microbial inoculations. On the contrary, the proline content parameter showed decreasing values with increasing rates of PG during the two growing seasons (Table 1). In addition, the combined application treatment (PG4 + T3) was the best treatment among all tested applications. For instance, in the 2019 season, the highest values of 2.73 mg g^−1^ FW and 0.970 µg g^−1^ FW were recorded for chlorophyll and carotenoid contents, respectively, while the PG3 + T3 treatment showed the lowest value (6.16 µmole g^−1^ FW) for proline content compared to the other treatments and the control (Table 1). A similar trend was observed in the 2020 season.

### 2.2. Antioxidant Enzyme Activities

**Figure 1:**
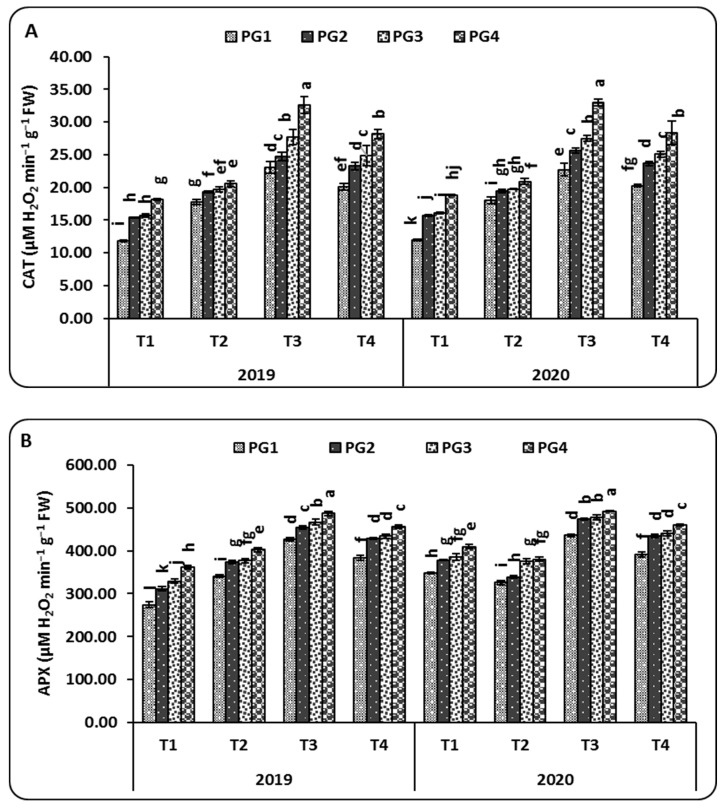

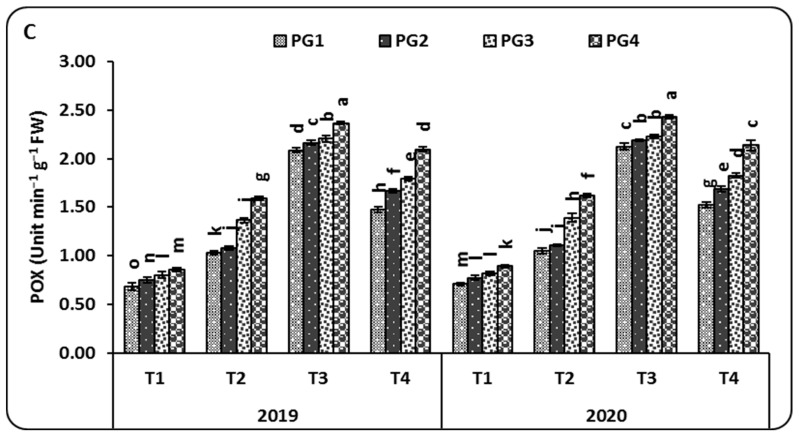
The combined effect of different rates of phosphogypsum and microbial inoculations on catalase (CAT) (**A**), ascorbate peroxidase (APX) (**B**), and peroxidase (POX) (**C**) activities in maize leaves grown in salt-affected soils during the 2019 and 2020 seasons. Means of the same growing season designated with different letters indicate significant differences among treatments according to the Duncan test (*p* < 0.05). Values are the means ± standard deviation (SD) from 3 replicates (means ± SD). PG1—0 t ha^−1^; PG2—3 t ha^−1^; PG3—6 t ha^−1^; PG4—9 t ha^−1^; T1—*A. lipoferum* (control); T2—*A. lipoferum* + *B. coagulans*; T3—*A. lipoferum* + *B. circulance*, and T4—*A. lipoferum* + *B. subtilis*.

The data presented in Figure 1 show that activities of catalase (CAT), ascorbate peroxidase (APX), and peroxidase (POX) were significantly changed due to the PG rates and microbial inoculation treatments at 60 days after sowing. The highest rate of PG added to the maize plants grown in salt-affected soil increased the activity of the CAT enzyme compared to the lowest addition rate. Interestingly, CAT activity is directly proportional to the increased rates of PG used in this study. Treated maize plants with PG4 (9 ton ha^−1^) significantly increased CAT activity (μM H_2_O_2_ g^−1^ FW min^−1^) from 20.07 (*A. lipoferum*, T1) to 23.27 (*A. lipoferum* + *B. coagulans*, T2), 24.82 (*A. lipoferum* + *B. circulance*, T3), and 28.18 (*A. lipoferum* + *B. subtilis*, T4) in the 2019 season, whereas in the 2020 season, the same rate of PG increased CAT activity from 20.25 (*A. lipoferum*, T1) to 23.66 (*A. lipoferum* + *B. coagulans*, T2), 25.07 (*A. lipoferum* + *B. circulance*, T3), and 28.32 (*A. lipoferum* + *B. subtilis*, T4) significantly, as shown in Figure 1A. From the results mentioned above, PG4 application and inoculation with *A. lipoferum* + *B. circulance* (T3) was the best treatment compared to other application treatments. In the same way, the application of PG and microbial inoculation alleviated the detrimental effect of salinity on the antioxidant capacity represented in APX activity. APX activity (μM H_2_O_2_ g^−1^ FW min^−1^) changed significantly from 383.67 (*A. lipoferum*, T1) to 428.67 (*A. lipoferum* + *B. coagulans*, T2), 434.00 (*A. lipoferum* + *B. circulance*, T3), and 456.00 (*A. lipoferum* + *B. subtilis*, T4) in the 2019 season. Likewise, in the 2020 season, the recorded APX activity was 391.67 (*A. lipoferum*, T1), 434.67 (*A. lipoferum* + *B. coagulans*, T2), 441.00 (*A. lipoferum* + *B. circulance*, T3), and 460.00 (*A. lipoferum* + *B. subtilis*, T4) when PG was applied with a rate of 9 ton ha^−1^. Among all treatments, the PG4 rate and T3 treatment had the highest values of APX activity (Figure 1B). A similar trend was observed for the POX activity, for which the PG4 rate with the T3 treatment was the best among all tested applications recorded, with 2.37 unit min^−1^ g^−1^ FW and 2.43 unit min^−1^ g^−1^ FW, respectively (Figure 1C).

### 2.3. Microbial Communities

**Figure 2:**
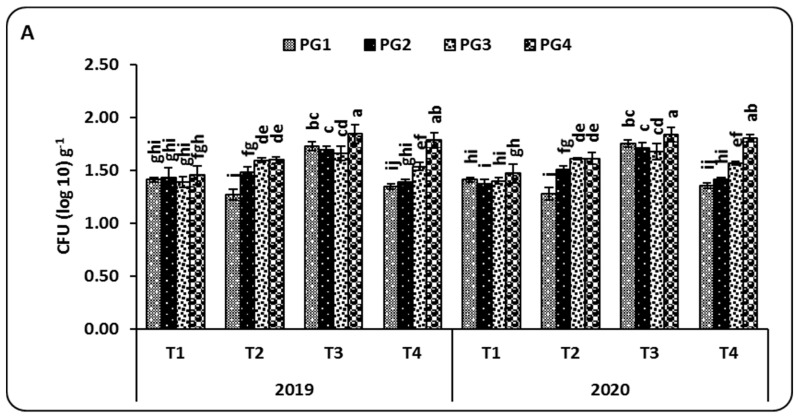

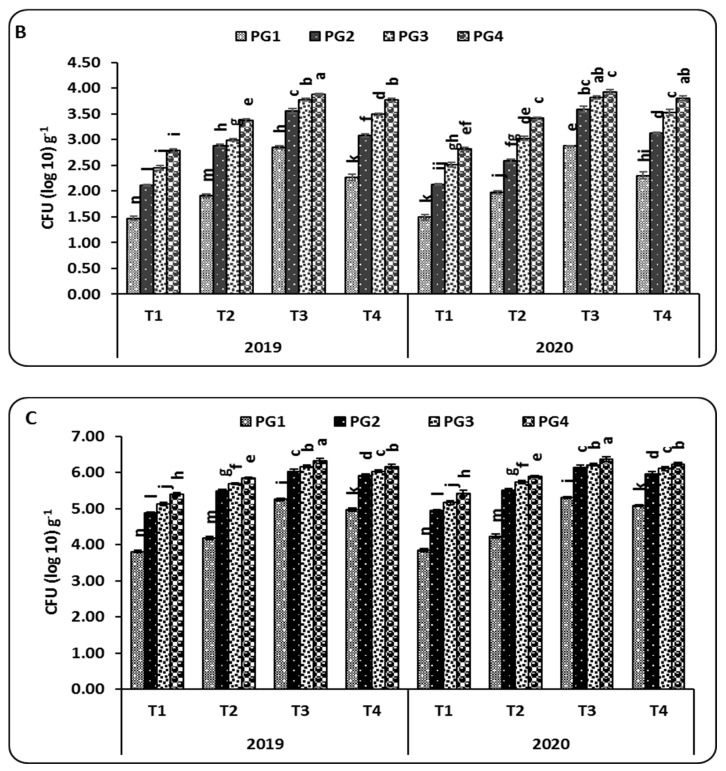
The combined effect of different rates of phosphogypsum and microbial inoculations on the total count of Azospirillum (**A**), the total count of Bacillus (**B**), and the total count of bacteria (**C**) in the rhizosphere of maize plants grown in salt-affected soils during the 2019 and 2020 seasons. Means of the same growing season designated with different letters indicate significant differences among treatments according to the Duncan test (*p* < 0.05). Values are the means ± standard deviation (SD) from 3 replicates (means ± SD). PG1—0 t ha^−1^; PG2—3 t ha^−1^; PG3—6 t ha^−1^; PG4—9 t ha^−1^; T1—*A. lipoferum* (control); T2—*A. lipoferum* + *B. coagulans*; T3—*A. lipoferum* + *B. circulance*, and T4—*A. lipoferum* + *B. subtilis*.

After two months from the time of sowing, the microbial communities, that is, the total count of *Azospirillum*, the total count of *Bacillus*, and the total count of bacteria in the rhizosphere of maize plants grown in salt-affected soil, were significant with respect to soil salinity and the application of PG and the microbial inoculation treatments (*p* < 0.05) in both the 2019 and 2020 seasons (Figure 2). 

In general, the results illustrate that different rates of PG attained differences in the microbial communities, showing a descending order of PG4 (9 ton ha^−1^) > PG3 (6 ton ha^−1^) > PG2 (3 t ha^−1^) > PG1 (0 t ha^−1^). On the other hand, the T3 treatment (*A. lipoferum* + *B. circulance*) showed the highest populations compared to the other treatments, with total counts of 1.85 and 1.84 CFU log10 g^−1^ of *Azospirillum* (Figure 2A), 3.88 and 3.92 CFU log10 g^−1^ of *Bacillus* (Figure 2B), and 6.32 and 6.37 CFU log10 g^−1^ of bacteria (Figure 2C) during the 2019 and 2020 seasons, respectively.

### 2.4. Soil Enzyme Activities

Dual inoculation with *Azospirillum* and different strains of *Bacillus* showed increases in the soil enzyme activity (dehydrogenase (DHA) and urease) in the rhizosphere of maize plants significantly with the single inoculation (*Azospirillum* only, control) under different application rates of phosphogypsum at 60 days after sowing (Table 2). Generally, dehydrogenase and urease activity was noted to increase with increasing phosphogypsum rates. The highest DHA activity, compared to other treatments, was 216.00 and 222.00 mg TPF g^−1^ soil day^−1^, followed by 190.00 and 195.00 mg TPF g^−1^ soil day^−1^ for the PG4T3 treatment (9 ton ha^−1^ and *A. lipoferum* + *B. circulance*) and the PG4T4 treatment (9 ton ha^−1^ and *A. lipoferum* + *B. subtilis*) during the 2019 and 2020 seasons, respectively (Table 2). On the other hand, under different application rates of PG, the T3 treatment (*A. lipoferum* + *B. circulance*) was the best inoculation treatment for urease enzyme activity, with recorded rates of 89.00, 97.00, 124.33, and 138.00 NH_4_^+^ − N g^−1^ soil day^−1^ in the 2019 season and 91.00, 99.00, 130.00, and 143.00 NH_4_^+^ − N g^−1^ soil day^−1^ in the 2020 season for 0, 3, 6, and 9 t ha^−1^ of PG, respectively. From the results mentioned above, PG4 and *A. lipoferum* + *B. circulance* treatment was the best treatment compared to other tested treatments (Table 2).

### 2.5. Macro-Elements, Na^+^ and K^+^/Na^+^ % in Maize Leaves 

Under different rates of PG, the inoculation of *Azospirillum* and their mixture with different strains of *Bacillus* (*B. coagulans*, *B. circulance*, and *B. subtilis*) led to increases in the percentages of the macroelements N, P, and K^+^ and the K^+^/Na^+^ ratio as well as decreases in the percentage of Na in maize leaves, with significant differences at 60 days after sowing (Table 3).

Under 9 t ha^−1^ of PG, the T3 treatment (seeds inoculated with *Azospirillum* + *B. circulance*) gave the highest percentages of N, P, and K compared to the control treatment (seeds inoculated with *Azospirillum* only), attaining increased rates of 26.3% and 29.5% for N, 55.1% and 50.9% for P and 74.3% and 71.5% for K during both the 2019 and 2020 seasons, respectively (Table 3). 

On the other hand, the percentage of Na^+^ decreased with the soil application of PG and microbial inoculation treatments. The greatest reduction in the percentage of Na^+^ was in maize leaves grown with the application of PG4 (9 t ha^−1^), which decreased from 1.73% (*A. lipoferum*, T1) to 1.75% (*A. lipoferum* + *B. coagulans*, T2), 0.85% (*A. lipoferum* + *B. circulance*, T3), and 1.39% (*A. lipoferum* + *B. subtilis*, T4) in the 2019 season, whereas in the 2020 season, the same rate of PG decreased the percentage of Na^+^ from 1.68% (*A. lipoferum*, T1) to 1.70% (*A. lipoferum* + *B. coagulans*, T2), 0.80% (*A. lipoferum* + *B. circulance*, T3), and 1.35% (*A. lipoferum* + *B. subtilis*, T4) significantly, as shown in (Table 3). In the same way, the T3 treatment (*A. lipoferum* + *B. circulance*) showed an increase in the K^+^/Na^+^ ratio, with recorded rates of 0.93% (0 t ha^−1^), 1.33% (3 t ha^−1^), 1.78% (6 t ha^−1^), and 2.31% (9 t ha^−1^) in the 2019 season; whereas in the 2020 season, the recorded rates were 0.97% (0 t ha^−1^), 1.38% (3 t ha^−1^), 1.91% (6 t ha^−1^), and 2.48% (9 t ha^−1^) compared to the other treatments under study (Table 3).

### 2.6. Micro-Elements in Maize Leaves

Field trials were carried out using a moderately salt-sensitive genotype of maize plants (*Zea mays* L., cv. HSC 10) in the presence of 0, 3, 6, and 9 t ha^−1^ of PG. The plants were exposed to different rates of PG, and when bacterized with dual-inoculation (*Azospirillum* + *Bacillus*), they showed significantly higher levels of plant micro-elements than single-inoculated plants (*Azospirillum* only) (Table 4). After 60 days from sowing, the PG rate (9 t ha^−1^) led to increases of 47.9, 20.08, 16.24, and 7.07% in maize leaves for Zn, Mn, Fe, and Cu (ppm), respectively, under the T3 treatment (*A. lipoferum* + *B. circulance*) compared to the control treatment during the 2019 season. A similar trend was observed in the 2020 season (Table 4). Thus, the PG4 rate (9 ton ha^−1^) showed the highest content of micro-elements (ppm) in maize plants than the other studied PG rates and the descending order of PG4 with microbial inoculation was as follows: PG4T3 (*A. lipoferum* + *B. circulance*) > PG4T4 (*A. lipoferum* + *B. subtilis*) > PG4T2 (*A. lipoferum* + *B. coagulans*) > PG4T1 (control) (Table 4). 

### 2.7. Soil Physicochemical Characteristics

**Figure 3:**
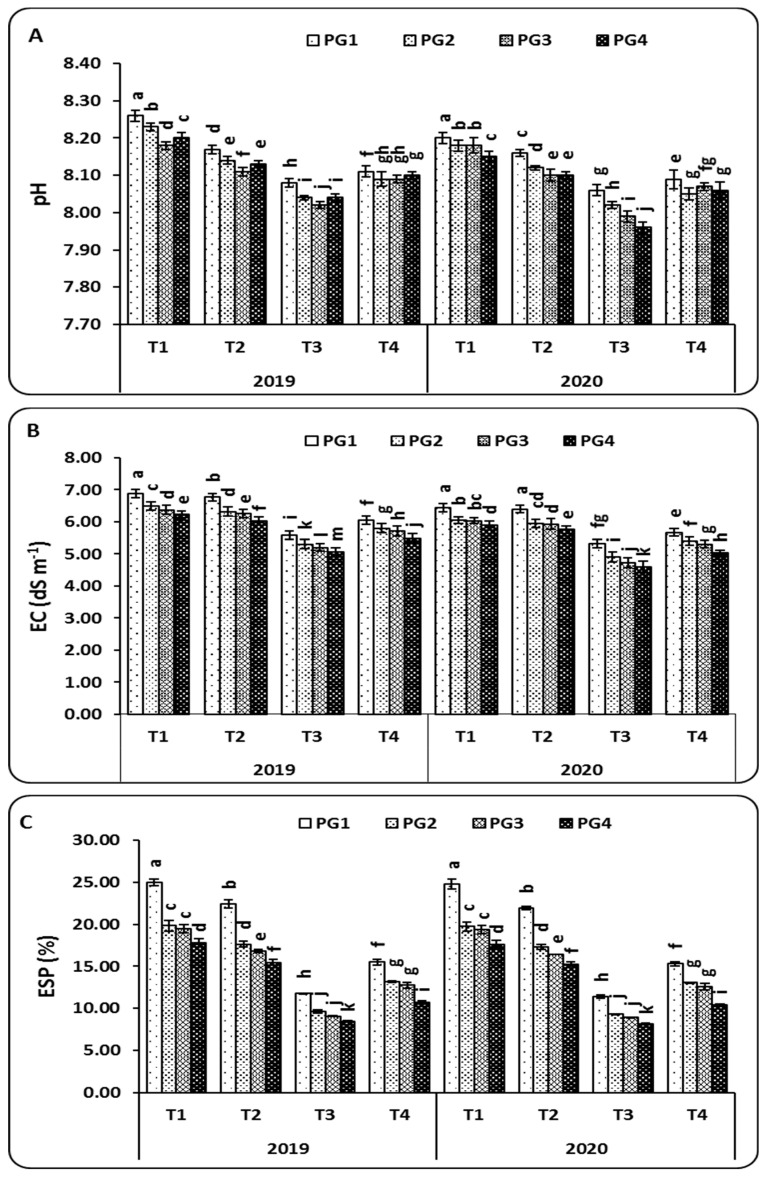
The combined effect of different rates of phosphogypsum and microbial inoculations on soil chemical characteristics pH (**A**), EC (**B**), and ESP (**C**) in salt-affected soils during the 2019 and 2020 seasons. Means of the same growing season designated with different letters indicate significant differences among treatments according to the Duncan test (*p* < 0.05). Values are the means ± standard deviation (SD) from 3 replicates (means ± SD). PG1—0 t ha^−1^; PG2—3 t ha^−1^; PG3—6 t ha^−1^; PG4—9 t ha^−1^; T1—*A. lipoferum* (control); T2—*A. lipoferum* + *B. coagulans*; T3—*A. lipoferum* + *B. circulance*, and T4—*A. lipoferum* + *B. subtilis*.

At harvest (120 DAS), and by comparing the initial soil traits, the applications of different PG rates and microbial inoculation treatments changed the physicochemical characteristics properties of the soil (Figure 3). It was found that the pH decremented gradually with increasing PG rates (PG2, PG3, and PG4). The highest decrease in pH was observed with the combined application of PG4 (9 t ha^−1^) and T3 (*A. lipoferum* + *B. circulance*) in both the 2019 and 2020 seasons. The use of the combined or individual applications of PG and microbial inoculation significantly improved the soil EC compared to the control plants in both the 2019 and 2020 seasons (Figure 3). The soil EC (dS m^−1^) changed significantly from 6.22 (*A. lipoferum*, T1) to 6.02 (*A. lipoferum* + *B. coagulans*, T2), 5.07 (*A. lipoferum* + *B. circulance*, T3), and 5.49 (*A. lipoferum* + *B. subtilis*, T4) in the 2019 season. Likewise, in the 2020 season, the recorded rates of soil EC were 5.89 (*A. lipoferum*, T1), 5.76 (*A. lipoferum* + *B. coagulans*, T2), 4.60 (*A. lipoferum* + *B. circulance*, T3), and 5.05 (*A. lipoferum* + *B. subtilis*, T4) when PG was applied at a rate of 9 t ha^−1^ (Figure 3). On the other hand, the combined treatment (PG4 + T3) was the best treatment for ESP, as the highest values of 8.44 and 8.18 % were recorded in the 2019 and 2020 seasons, respectively, as compared to the other treatments (Figure 3).

### 2.8. Maize Productivity

The results (Table 5) revealed that inoculation treatments in combination with PG showed significant influence on grains yield (kg ha^−1^) and yield-related parameters (ear length, ear diameter, grains/ear, and 100-grain weight), under salt-affected soil conditions during the two growing seasons. 

The combination treatment (*A. lipoferum* + *B. circulance*) with PG4 (9 ton ha^−1^) caused the maximum values of ear length (cm), ear diameter (cm), and grains/ear, recorded as 24.20, 4.70 and 458.00, respectively, compared to the single inoculation with the PG4 treatment (control), recorded as 18.23, 4.33, and 433.33 in the 2019 season, respectively. Likewise, in the 2020 season, the same trend was observed (Table 5).

In regard to the 100-grain weight, a different increase rate was noticed between the dual microbial inoculation treatments under the PG4 rate (9 t ha^−1^) compared to the control (single inoculation), with recorded rates of 1.63% and 1.60% with the T2 treatment (*A. lipoferum* + *B. coagulans*), 16.68% and 16.91% with the T3 treatment (*A. lipoferum* + *B. circulance*), and 8.31% and 8.36% with the T4 treatment (*A. lipoferum* + *B. subtilis*) during the 2019 and 2020 seasons, respectively (Table 5). On the other hand, a positive effect was caused by the combined application treatment (PG4 + T3) on the grain yields of maize plants, which were 6235.33 and 6309 kg ha^−1^ during the 2019 and 2020 seasons, respectively, compared to the other inoculation treatments and different rates of PG (Table 5). 

From the results mentioned above, the descending order for the microbial inoculations was T3 > T2 > T4 > T1, while the descending order of the PG rates was PG4 > PG3 > PG2 > PG1.

## 3. Discussion

In arid and semi-arid regions like Egypt, soil salinity problems have been reported to have a negative impact on soil quality and crop growth [29]. Indeed, saline conditions affect plant growth in two phases. In the first phase, inhibition occurs mainly through the lack of water availability due to the high concentration of the soil solution, and if salt stress continues for a long time, ion toxicity is the main factor that limits the plant metabolism and survival in a second phase [30,31,32].

The present study aimed to reduce the harmful effect of salt stress on maize plants by single and dual microbial inoculations (*A. lipoferum*, *A. lipoferum* + *B. coagulans*, *A. lipoferum* + *B. circulance*, and *A. lipoferum* + *B. subtilis*). Physiological traits, antioxidant enzymes, and physicochemical and biological activities of the soil, as well as nutrient contents and yield, were positively affected when maize plants were exposed to 3, 6, and 9 t ha^−1^ of PG combined with microbial inoculation in salt-affected soil during the 2019 and 2020 seasons.

### 3.1. Physiological Characteristics in Maize Leaves

It has been proven in previous studies that maize plants are more sensitive to the harmful effect of salinity, which leads to decreases in photosynthetic pigments and physiological properties due to a lack of biosynthesis. Therefore, the improvement in the physiological traits of maize plants grown under salinity stress was lower than those produced with the combined application of PG and microbial inoculation (Table 1). Maize plants exposed to salinity stress (with amendments of PG and microbial inoculation) resulted in increases in chlorophyll and carotenoids and a decrease in proline content, which is attributed to an increase in soil water availability. When using the synergistic effect of PG and microbial inoculation, it increased the available soil water, reduced osmotic stress, and avoided turgor loss under water stress in salt-affected soils compared to control plants (neither PG nor microbial inoculation) [29,33,34]. In addition, maize plants inoculated with *A. lipoferum* + *B. circulation* with PG4 (9 t ha^−1^) led to an amplification of root hydraulic conductivity, which was positively reflected in the chlorophyll pigments compared to untreated plants when exposed to salinity stress [17,35]. 

### 3.2. Antioxidant Enzyme Activities

Plants have many antioxidant strategies to reduce toxic compounds. Thus, enhancing the antioxidant defense in plants can lead to increased tolerance to various stress factors. Additionally, electrolyte leakage is a key indicator of cell membrane permeability of plants under abiotic stresses, such as salinity, which firstly target the cell membrane [36]. Therefore, the highest activity of antioxidant enzymes alleviates the adverse effect of salinity, especially on cell membrane permeability, through lowering the lipid peroxidation. In addition, to prevent oxidative damage under soil salinity stress conditions, antioxidant enzymatic activities in plants are essential for dealing with the harmful effects of reactive oxygen species (ROS) [29,37,38]. From our results, antioxidant enzymatic activities (CAT, APX, and POD) were significantly increased with increased rates of PG and microbial inoculation (Figure 1). Under 9 t ha^−1^ of PG, the highest CAT, APX, and POD activities were attained in the maize plants exposed to the T3 treatment (*A. lipoferum* + *B. circulation*), followed by the plants that were exposed to the T4 treatment (*A. lipoferum* + *B. subtilis*) and the plants that were exposed to the T2 treatment (*A. lipoferum* + *B. coagulans*) (Figure 1). 

These findings are supported by [39], suggesting that wheat plants inoculated with *B. phytofirmans* strain PsJN could be effectively used to improve the growth and increase the enzyme activities under abiotic stresses. Additionally, [40] suggested that inoculation with *A. lipoferum* could protect wheat plants from the harmful effects of abiotic stresses through changes in the antioxidant defense system. Smaoui-Jardak [41] reported that PG doses not exceeding 20% may have caused increasing levels of enzyme activities (CAT, GPX, APX, MDHAR, DHAR, and GR) in tomato plants in saline soil.

### 3.3. Microbial Communities and Soil Enzyme Activities

In soil, microbial biomass and microbial activity play significant roles in the decomposition of organic matter and the maintenance of soil nutrients [42,43]. From the obtained results (Figure 2 and Table 2), it can be seen that applying PG to saline soil has different effects on the total counts of *Azospirillum*, *Bacillus*, and bacteria, as well as on soil enzyme activities (DHA and urease), depending on inoculation treatments and different application rates of PG. 

Applying PG (9 t ha^−1^) with the T3 treatment (*A. lipoferum* + *B. circulation*) significantly increased the microbial biomass and soil enzyme activities due to their effects on increasing the levels and availability of soil nutrient contents, as well as increasing soluble organic compounds, which are able to support soil fertility. 

These findings are supported by some other researchers; [43] showed that soil amended with 10% PG significantly improved microbial growth (total counts of bacteria and fungi) and soil enzyme activities (invertase, amylase, and cellulase) compared to different rates of PG. Al-Enazy [15] reported that the highest values of total soil microbial counts were observed in maize plants inoculated with *Azotobacter chroococcum*, *Bacillus megaterium* var. *phosphaticum*, and *Pseudomonas fluorescens* and treated with 30 g PG kg^−1^. Mahmoud [28] demonstrated that the soil microbial biomass, CO_2_ evolution, and dehydrogenase activity (DHA) increased significantly in maize plants with the application of PG (10 t ha^−1^) with nitrogen fertilizer (285 kg N ha^−1^).

### 3.4. The Percentages of Macro-Elements, Na^+^, and K^+^/Na^+^ in Maize Leaves 

The addition of PG with microbial inoculation to saline soil can increase the availability of N, P, and K nutrient cycling in the soil and increase crop production [15]. In the current study (Table 3), the results show that the leaves of inoculated maize plants grown in saline soil had significantly higher macro-nutrient (N, P, and K) content than uninoculated plants, especially with the synergistic application of PG (9 t ha^−1^) with microbial inoculation (*A. lipoferum* + *B. circulation*). In addition, the Na^+^ content in maize plants was reduced, while the K^+^ content was augmented, resulting in an improved K^+^/Na^+^ ratio. In line with our findings, [44] showed that applying soil ameliorants (gypsum and sulfur) with appropriate rhizobial strains is a very important practice to enhance N uptake in cowpea plants grown in saline soils. Additionally, [15] reported that maize plants grown in saline soils and treated with PG50 (50 g kg^−1^) with co-inoculation (*Azotobacter*, *Bacillus megaterium*, and *Pseudomonas*) increased N uptake (98.32 mg plant^−1^), compared to the control treatment (51.53 mg plant^−1^). Similarly, decreasing the content of Na^+^ ions in the leaves of maize plants inoculated with PGPR (*Azospirillum lipoferum* + *Bacillus circulance*) under saline soil might be due to the exopolysaccharide produced by PGPR, which binds the Na^+^ ions in the soil, leading to a decrease in its uptake [17].

### 3.5. Micro-Elements in Maize Leaves

There are many microelements (Fe, Zn, Mn, Cu, and Mo) that are essential for plant nutrition, and they are needed in small quantities [45,46]. According to [47], these elements are very important to plants, as they participate in the composition of proteins, DNA, enzymes, amino acids, and photosynthetic processes. In the same context, soil microorganisms can improve plant nutrients by microelements through various methods using appropriate traits that identify them as plant growth promoters [46,48].

The synergistic application of PG (9 t ha^−1^) with co-inoculated plants significantly increased the concentrations of microelements compared to single inoculation (Table 4). These results are consistent with the findings of [49], who reported that soil inoculated with mycorrhizal fungi and amended with PG enhanced the element content and growth of wheat plants. In addition, [15] showed that PG along with co-inoculation may be an important approach for ameliorating the negative results of salinity on the plant growth of maize under saline soil conditions.

### 3.6. Soil Physicochemical Characteristics

The application of PG alone or with microbial inoculation treatments caused a significant decrease in soil pH compared to the control treatment (Figure 3). This decrease can be attributed to the acidifying effect of residual sulfuric acid in PG [26,49]. These results are in agreement with those of [43,50], showing that soil pH tends to decrease with increasing rates of application of a PG amendment. Although the addition of PG did not have a significant effect on the soil EC and ESP, it is interesting to note that the co-inoculation of bacteria along with 0, 3, 6, and 9 t ha^−1^ resulted in significant decreases in the EC and ESP values compared to the control (Figure 3). This decrease in soil EC could be explained by the production of exogenous polysaccharides (EPS) by plant growth-promoting rhizobacteria (PGPR), which are able to bind cations, including Na^+^, and thus, alleviate salt stress in plants grown under saline conditions. Similar to our finding, [51] reported that the co-inoculation of PGPR, including *Pseudomonas moraviensis* and *B. cereus*, significantly reduced soil EC. Al-Enazy [14] found that the co-inoculation of maize plants with PGPR, including *A. chroococcum*, *B. megaterium* var. *phosphaticum*, and *P. fluorescens*, and treated with 50 g PG kg^−1^ significantly reduced soil EC. Additionally, [28] showed that the pH and EC decreased significantly (*p* < 0.05) with increased PG application rates. For example, PG at a rate of 10 ton ha^−1^ in clay soil with maize plants showed a higher reduction in EC, ranging from 26% with the recommended nitrogen fertilizer (NF) with the water treatment residual at 5 t ha^−1^ (WTR5) to 48% for NF+PG10 compared with the other treatments. 

### 3.7. Maize Productivity

The grain yield and yield-related parameters of maize plants grown in saline soils treated with 9 t ha^−1^ and inoculated with *A. lipoferum* + *B. circulation* increased as compared to the other studied treatments (Table 5). The addition of PG to the soil had positive effects on the maize yield because of its high content of P, Ca, and S, as well as the improvement of the physicochemical properties of the soil.

These findings are supported by previous studies; [27] demonstrated a positive effect of PG amendment on the biological and chemical properties of soil and cabbage yield in China. Michalovic [52] showed increased productivity of maize plants with PG additions. Smaoui-Jardak [41] suggested that PG application (20%) seems to be beneficial for increasing the productivity of tomato plants under saline soil conditions. Mahmoud et al. [28] reported that the use of 10 t ha^−1^ of PG was optimal for achieving high productivity of maize plants grown in clay soils. 

## 4. Materials and Methods

### 4.1. Phosphogypsum (PG)

From a fertilizer industry factory in El-Sharkia Governorate, Egypt, phosphogypsum (PG) was brought to the laboratory and ground, and then passed through a 2-mm screen. The initial physical and chemical properties of PG are as follows: the contents of Ca^2+^ (meq L^−1^), Mg^2+^ (meq L^−1^), O.M (%), and CEC (cmol Kg^−1^) were 28.55, 3.65, 5.68, and 59.87, respectively. The pH and EC were 3.65, and 3.95 dS m^−1^, respectively. The pH was measured with a digital pH meter, and the EC was measured by a conductivity meter in a 1:5 soil/water ratio [53]. The total contents of S, P, K, Al, and Cd in the PG were 14.3%, 2.32%, 0.09%, 0.13%, and 2.12%, respectively, which were measured by coupled plasma–optical emission spectrometry (ICP-OES) (PerkinElmer Optima 4300 DV).

### 4.2. Microorganisms and Culture Conditions 

One strain of *Azospirillum lipoferum* SP2 and three strains of *Bacillus* (*B. coagulans* NCAIM B.01123, *B. circulance* NCAIM B.02324, and *B. subtilis* MF497446) were obtained from Bacteriology Laboratory, Sakha Agricultural Research Station, Kafr El-Sheikh, Egypt. The standard culture conditions were prepared with semi-solid malate medium for *A. lipoferum* [54], and with nutrient broth medium for the *Bacillus* strains [55]. 

### 4.3. Experimental Setup and Treatments

In a split-plot design with three replicates, a field experiment was performed in salt-affected soil during the two summer growing seasons at the Sakha Agricultural Research Station, Kafr El-Sheikh Governorate, Egypt, to investigate the application of PG combined with microbial inoculation on chemical and biological soil properties, plant physiology, antioxidant enzymes, macro- and micronutrient contents, and the yield of a moderately salt-sensitive genotype of maize plants (*Zea mays* L., cv. HSC 10). The main plots were divided into four groups of different PG rates (0, 3, 6, and 9 t ha^−1^), and the sub-plots (42 m^2^, 10 rows, 7 m long, and 0.70 m apart) were divided by the microbial inoculation treatments (*A. lipoferum* (control), *A. lipoferum* + *B. coagulans*, *A. lipoferum* + *B. circulance*, and *A. lipoferum* + *B. subtilis*). The physicochemical and biological properties of the experimental area are presented in Table 6, and the meteorological data are tabulated in Table 7. During the seedbed preparation, phosphorus fertilizer was applied at a rate of 480 kg ha^−1^ as calcium superphosphate (15.5% P_2_O_5_) and 120 kg ha^−1^ as potassium sulfate (48% K_2_O), in one dose, while the different rates of PG were incorporated into the soil surface (0–20 cm) as the final step of seedbed preparation. Nitrogen fertilizer was applied at a rate of 290 Kg ha^−1^ as urea (46.5% N), in equal two doses (at 20 and 40 days of sowing). The seeds of maize were provided by the Maize Research Department, Sakha, Kafr El-Sheikh, Egypt. The seeds were planted on May 7th and 5th in the summer seasons of 2019 and 2020, respectively, at a rate of 33 kg ha^−1^, and then thinned to one plant per hill before the first irrigation. The inoculation treatments were prepared as peat-based inoculums, with 30 mL of 10^9^ CFU mL^−1^ from each culture per 60 g of sterilized carrier, and mixed carefully with the maize seeds before sowing. The plants were irrigated every 12 days and all recommended agricultural practices were followed through the growing seasons according to the Ministry of Agriculture, Egypt.

### 4.4. Measurements and Analyses

#### 4.4.1. Physiological Characteristics in Maize Leaves

In the fourth topmost fully expanded leaves, the total chlorophyll, carotenoid, and proline concentrations were determined 60 days after sowing. Briefly, 0.5 g of fresh leaf was homogenized in 1 mL of acetone (100%) for 48 h at 4 °C, centrifuged for 10 min at 5000× *g*, and the total chlorophyll and carotenoid were calculated according to [57]. For the proline content, 5 mL of ethanol (95%) and 0.2 g of fresh leaf were homogenized and centrifuged at 5000× *g*. The supernatant was collected for estimation. Under boiling water bath conditions (100 °C), a mix of 1 mL of alcoholic extract, 1 mL of dH_2_O, 2 mL of ninhydrin, and 2 mL of glacial acetic acid was added to test tubes. After 1 h, the reaction was stopped in cold water and mixed with 4 mL of toluene, and then estimated at 520 nm using a UV spectrophotometer (Model 6705). Using a standard curve, the proline concentration was expressed as µmol g^−1^ FW of leaves [58]. 

#### 4.4.2. Assay of Antioxidant Enzyme Activities 

After two months from the date of sowing, the antioxidant enzymes were determined. The reaction contained 50 mM Na^+^ of phosphate buffer (pH 7.0) + 20 µL/mL enzymatic extract + 1 mM H_2_O_2_. At 240 nm, the catalase (CAT) activity (μM H_2_O_2_ min^−1^ g^−1^ FW) was determined according to the methods of [59]. For ascorbate peroxidase (APX) activity, 2 mL of extraction buffer (90 mM Na_2_HPO_4_ buffer (pH 7.8) with 8% glycerol, 1 mM EDTA, and 5 mM ascorbate was homogenized with 0.1 g fresh leaf. Polyvinyl pyrrolidone (0.3 g/g tissue) was added, and it was then centrifuged at 10,000× *g* for 10 min at 4 °C. APX activity (μM H_2_O_2_ min^−1^ g^−1^ FW) was determined at 290 nm by adding 200 μL of enzyme extract with 25 mM of phosphate buffer (pH 7.0), 0.1 mM of EDTA, 0.25 mM of ascorbic acid, and 1.0 mM of H_2_O_2_ [60]. At 420 nm, peroxidase (POX) activity (Unit min^−1^ g^−1^ FW) was determined by the technique of [61]. The reaction contained 50 mM of phosphate buffer (pH 7.0), 28 µL of guaiacol, 100 µL of enzymatic extract, and 19 µL of H_2_O_2_. 

#### 4.4.3. Microbial Community Estimations

In the rhizosphere of soil samples, 10 g was transferred into a glass bottle containing 90 mL of sterile distilled water and shaken for 0.5 h at 150 rpm. According to [54], the most probable number method was used for the total count of *Azospirillum* by semi-solid malate medium and calculated using the methods of [62]. The total count of *Bacillus* was estimated by nutrient agar medium [55] after pasteurizing the soil dilutions (10^−1^–10^−3^). According to the methods of [63], the total count of bacteria was estimated using soil extract agar media. All microbial populations were expressed as the CFU (log 10) g^−1^ dry soil at 60 days after sowing.

#### 4.4.4. Soil Enzyme Activities

Dehydrogenase (DAH) activity in the soil samples was estimated according to [64]. Briefly, in test tubes, 10 g of soil sample was mixed with 0.2 g CaCO_3_. Then, 1 mL of 2,3,5-triphenyl tetrazolium chloride (TTC, 3%), 1 mL of glucose solution (1%), and 8 mL of dH_2_O were added and then incubated at 30 °C for 24 h after the mixture was stoppered with a rubber cork. After incubation, the contents of the test tubes were rinsed down into a small beaker and a slurry was made by adding 10 mL of methanol. It was then filtered by using Whatman No. 50 filter paper. At 485 nm, the intensity of the red color was measured against a methanol blank using a UV/visible spectrophotometer (Model 6705). By using a standard curve of formazan, the results are expressed as the mg TPF g^−1^ soil day^−1^.

As adopted from [65], the urease activity of the soil sample was determined. Briefly, in 100 mL-capacity Erlenmeyer flasks, 5 g of the soil sample was mixed with 0.5 mL of toluene for 15 min. Then, 10 mL of phosphate buffer (pH 7.6) and 10 mL of 10% urea solution were added and then shaken for 5 min and incubated at 30 °C for 24 h. The contents of the flasks were filtered through Whatman No. 42 filter paper after incubation, and the remaining soil sample in the flask had 15 mL of KCl solution added (1 N), and it was shaken for 5 min before being filtered again; the total filtrate was made up to 100 mL. In a 50 mL volumetric flask, 1 mL filtrate was mixed with 1 mL of sodium and potassium tartrate (10%). Then, 1 mL of gum acacia solution (1%) and 5 mL of Nesslers reagent were added, supplementing the volume to 50 mL with distilled water [66]. Using a UV/visible spectrophotometer (Model 6705), the yellow color was measured at 410 nm. Using a standard curve of (NH_4_)_2_SO_4_ solution, the results were expressed as the mg NH_4_^+^ − N g^−1^ soil day^−1^. 

#### 4.4.5. Determinations of Macro and Micro-Nutrients in Maize Leaves

At 60 days after sowing, 0.5 g of ground leaf sample (the fourth topmost fully expanded leaves) were digested on a hot plate using concentrated sulfuric acid and 30% H_2_O_2_ according to the methods of [67]. The nitrogen content (mg plant^−1^) was determined by micro-Kjeldahl, as described by [68]. The phosphorus content (mg plant^−1^) was estimated spectrophotometrically by the methods of [69]. The Na^+^, K^+^, and the K^+^/Na^+^ ratio were determined by a Flame photometer according to the methods of [70]. In addition, the micronutrient contents (mg plant^−1^) of Zn, Mn, Fe, and Cu were measured using an atomic adsorption spectrophotometer (Perkin Elmer 3300) according to the methods of [70]. 

#### 4.4.6. Soil Chemical Characteristics

Using an augur at 120 days from sowing (harvest), soil samples (30 cm depth) were collected and homogenized as a single sample per replicate. The soil samples were dried in open air, ground, and passed through a 2-mm sieve. The pH was estimated in a 1:2.5 suspension (soil/distilled water) by a pH meter (Genway, UK). In the soil paste extract, the EC (dS m^−1^) was determined using an EC meter (Genway, UK). Based on the equation by [71], the exchangeable sodium percentage (ESP) was measured. 

#### 4.4.7. Maize Productivity

After four months from sowing and at a 15.5% moisture content, the harvest was performed and 10 plants were randomly collected from the fourth inner ridges to estimate yield traits such as the ear length (cm), ear diameter (cm), number of grains per ear, 100-grain weight, and the grain yield (kg ha^−1^). 

### 4.5. Statistical Analysis

Using a split-plot analysis of variances (ANOVA) and SPSS 20.0 software, the data were analyzed. Duncan’s multiple range test was used for comparison among the treatment means [72]. 

## 5. Conclusions

The results of the present study show that maize plant growth and nutrient uptake can be increased by using PG along with bacterial co-inoculation under saline soil conditions. This technique (the combination of PG and microbial inoculation) is considered to be a promising technique for mitigating the harmful effects of soil salinity on plant growth, and this is reflected in an increase in yield. Therefore, the results suggest that applying a PG (9 t ha^−1^) and co-inoculation (*A. lipoferum* + *B. circulance*) treatment can significantly increase the plant physiology, antioxidant enzymes, microbial activity, nutrient uptake, and productivity of maize plants under saline soil conditions. 

## Figures and Tables

**Table 1:** The combined effect of different rates of phosphogypsum and microbial inoculations on total chlorophyll, carotenoids and proline contents in maize leaves grown in salt-affected soils during the 2019 and 2020 seasons.

Year	Phosphogypsum Rates (PG)	Inoculation (I)	Total Chlorophyll (mg g^−1^ FW)	Carotenoids (μg g^−1^ FW)	Proline (µmol g^−1^ FW)
**2019**	PG1	T1	1.00 ± 0.04 ^j^	0.297 ± 0.01 ^l^	9.94 ± 0.15 ^a^
		T2	1.16 ± 0.05 ^i^	0.377 ± 0.01 ^j^	9.60 ± 0.20 ^b^
		T3	1.29 ± 0.02 ^h^	0.357 ± 0.01 ^jk^	8.79 ± 0.20 ^cd^
		T4	1.25 ± 0.03 ^h^	0.330 ± 0.02 ^kl^	8.97 ± 0.20 ^c^
	PG2	T1	1.74 ± 0.07 ^g^	0.540 ± 0.07 ^i^	8.77 ± 0.14 ^cd^
		T2	1.91 ± 0.02 ^f^	0.710 ± 0.02 ^g^	8.30 ± 0.20 ^e^
		T3	2.16 ± 0.04 ^d^	0.730 ± 0.03 ^fg^	7.49 ± 0.20 ^ghi^
		T4	1.90 ± 0.03 ^f^	0.693 ± 0.04 ^gh^	7.68 ± 0.20 ^g^
	PG3	T1	1.85 ± 0.03 ^f^	0.653 ± 0.03 ^h^	8.59 ± 0.05 ^d^
		T2	2.06 ± 0.04 ^e^	0.867 ± 0.04 ^d^	7.53 ± 0.25 ^gh^
		T3	2.47 ± 0.05 ^b^	0.883 ± 0.03 ^cd^	6.16 ± 0.08 ^k^
		T4	2.07 ± 0.04 ^e^	0.820 ± 0.02 ^e^	7.27 ± 0.05 ^ij^
	PG4	T1	2.21 ± 0.03 ^d^	0.767 ± 0.02 ^f^	8.13 ± 0.10 ^ef^
		T2	2.31 ± 0.03 ^c^	0.927 ± 0.02 ^b^	8.08 ± 0.05 ^f^
		T3	2.73 ± 0.05 ^a^	0.970 ± 0.01 ^a^	7.21 ± 0.25 ^j^
		T4	2.17 ± 0.03 ^d^	0.923 ± 0.01 ^bc^	7.45 ± 0.05 ^hi^
**2020**	PG1	T1	1.04 ± 0.04 ^k^	0.32 ± 0.02 ^k^	9.95 ± 0.03 ^a^
		T2	1.20 ± 0.05 ^j^	0.40 ± 0.03 ^i^	9.67 ± 0.11 ^b^
		T3	1.33 ± 0.03 ^i^	0.38 ± 0.01 ^ij^	8.84 ± 0.08 ^c^
		T4	1.29 ± 0.02 ^i^	0.35 ± 0.01 ^jk^	8.91 ± 0.10 ^c^
	PG2	T1	1.80 ± 0.07 ^h^	0.57 ± 0.06 ^h^	8.84 ± 0.07 ^c^
		T2	1.92 ± 0.02 ^g^	0.73 ± 0.02 ^f^	8.19 ± 0.16 ^e^
		T3	2.19 ± 0.04 ^e^	0.75 ± 0.03 ^f^	7.51 ± 0.14 ^g^
		T4	1.92 ± 0.02 ^g^	0.72 ± 0.04 ^f^	7.88 ± 0.04 ^f^
	PG3	T1	1.89 ± 0.04 ^g^	0.67 ± 0.03 ^g^	8.66 ± 0.05 ^d^
		T2	2.11 ± 0.04 ^f^	0.89 ± 0.06 ^c^	7.52 ± 0.21 ^g^
		T3	2.50 ± 0.05 ^b^	0.90 ± 0.04 ^c^	6.17 ± 0.05 ^j^
		T4	2.10 ± 0.05 ^f^	0.85 ± 0.03 ^d^	7.25 ± 0.08 ^h^
	PG4	T1	2.26 ± 0.04 ^d^	0.79 ± 0.02 ^e^	8.10 ± 0.05 ^e^
		T2	2.34 ± 0.04 ^c^	0.95 ± 0.02 ^b^	8.08 ± 0.04 ^e^
		T3	2.76 ± 0.06 ^a^	1.00 ± 0.01 ^a^	6.98 ± 0.11 ^i^
		T4	2.21 ± 0.02 ^de^	0.95 ± 0.02 ^b^	7.39 ± 0.05 ^gh^
**F-test**					
Phosphogypsum rates (PG)			***	***	***
Inoculation (I)			***	***	***
Interaction (PG × I)			***	***	***

Means of the same growing season designated with different letters indicate significant differences among treatments according to the Duncan test (*p* < 0.05). Values are the means ± standard deviation (SD) from 3 replicates (mean ± SD). PG1—0 t ha^−1^; PG2—3 t ha^−1^; PG3—6 t ha^−1^; PG4—9 t ha^−1^; T1—*A. lipoferum* (control); T2—*A. lipoferum* + *B. coagulans*; T3—*A. lipoferum* + *B. circulance*, and T4—*A. lipoferum* + *B. subtilis*; ***—High significant.

**Table 2:** The combined effect of different rates of phosphogypsum and microbial inoculations on soil enzyme activity (dehydrogenase and urease) in the rhizosphere of maize plants grown in salt-affected soils during the 2019 and 2020 seasons.

Year	Phosphogypsum Rates (PG)	Inoculation (I)	DHA (mg TPF g^−1^ Soil d^−1^)	Urease (NH_4_^+^ − N g^−1^ Soil Day^−1^)
**2019**	PG1	T1	50.00 ± 4.58 ^l^	45.33 ± 1.53 ^j^
		T2	74.00 ± 2.65 ^k^	58.33 ± 3.51 ^i^
		T3	80.00 ± 3.00 ^jk^	89.00 ± 3.61 ^e^
		T4	84.00 ± 4.00 ^j^	71.33 ± 3.79 ^h^
	PG2	T1	104.67 ± 5.51 ^i^	57.00 ± 2.65 ^i^
		T2	147.33 ± 5.69 ^f^	71.00 ± 2.00 ^h^
		T3	184.33 ± 4.16 ^c^	97.00 ± 2.00 ^d^
		T4	169.33 ± 5.51 ^d^	82.67 ± 3.21 ^f^
	PG3	T1	122.33 ± 5.51 ^h^	68.67 ± 2.52 ^h^
		T2	158.33 ± 4.04 ^e^	77.67 ± 3.51 ^g^
		T3	201.33 ± 3.51 ^b^	124.33 ± 3.51 ^b^
		T4	176.00 ± 3.61 ^d^	92.67 ± 3.51 ^e^
	PG4	T1	134.33 ± 4.16 ^g^	76.33 ± 2.08 ^g^
		T2	172.00 ± 4.58 ^d^	89.67 ± 3.06 ^e^
		T3	216.00 ± 5.57 ^a^	138.00 ± 4.58 ^a^
		T4	190.00 ± 3.61 ^c^	106.00 ± 4.36 ^c^
**2020**	PG1	T1	51.67 ± 3.51 ^n^	46.67 ± 2.08 ^l^
		T2	77.67 ± 0.58 ^m^	61.33 ± 1.53 ^k^
		T3	81.67 ± 3.21 ^m^	91.00 ± 3.46 ^f^
		T4	87.00 ± 2.65 ^l^	76.00 ± 3.61 ^i^
	PG2	T1	111.00 ± 2.65 ^k^	60.33 ± 4.73 ^k^
		T2	151.33 ± 3.51 ^h^	72.33 ± 3.21 ^j^
		T3	187.00 ± 3.61 ^d^	99.67 ± 2.08 ^d^
		T4	172.33 ± 5.69 ^f^	82.67 ± 1.15 ^g^
	PG3	T1	129.67 ± 1.53 ^j^	70.33 ± 3.51 ^j^
		T2	162.00 ± 3.61 ^g^	80.00 ± 4.58 ^gh^
		T3	209.67 ± 1.15 ^b^	130.00 ± 3.61 ^b^
		T4	179.00 ± 4.00 ^e^	95.00 ± 4.00 ^e^
	PG4	T1	138.33 ± 4.04 ^i^	78.67 ± 1.53 ^hi^
		T2	175.33 ± 3.79 ^ef^	91.67 ± 3.51 ^ef^
		T3	222.67 ± 5.51 ^a^	143.33 ± 4.51 ^a^
		T4	195.00 ± 6.08 ^c^	112.67 ± 5.69 ^c^
**F-test**	Phosphogypsum rates (PG)		***	***
	Inoculation (I)		***	***
	Interaction (PG × I)		***	***

Means of the same growing season designated with different letters indicate significant differences among treatments according to the Duncan test (*p* < 0.05). Values are the means ± standard deviation (SD) from 3 replicates (means ± SD). PG1—0 t ha^−1^; PG2—3 t ha^−1^; PG3—6 t ha^−1^; PG4—9 t ha^−1^; T1—*A. lipoferum* (control); T2—*A. lipoferum* + *B. coagulans*; T3—*A. lipoferum* + *B. circulance*, and T4—*A. lipoferum* + *B. subtilis*; ***—High significant.

**Table 3:** The combined effect of different rates of phosphogypsum and microbial inoculations on the percentages of N, P, K, and Na and the K/Na ratio in maize leaves grown in salt-affected soils during the 2019 and 2020 seasons.

Year	Phosphogypsum Rates (PG)	Inoculation (I)	N	P	K	Na	K/Na
**2019**	PG1	T1	1.03 ± 0.03 ^l^	0.24 ± 0.02 ^l^	0.98 ± 0.03 ^k^	2.18 ± 0.04 ^a^	0.44 ± 0.02 ^m^
		T2	1.07 ± 0.04 ^l^	0.26 ± 0.02 ^k^	1.00 ± 0.02 ^k^	2.08 ± 0.04 ^b^	0.48 ± 0.02 ^lm^
		T3	1.34 ± 0.02 ^h^	0.37 ± 0.02 ^i^	1.33 ± 0.03 ^ef^	1.43 ± 0.02 ^j^	0.93 ± 0.01 ^e^
		T4	1.14 ± 0.03 ^k^	0.34 ± 0.02 ^j^	1.25 ± 0.01 ^g^	1.71 ± 0.03 ^fg^	0.73 ± 0.02 ^g^
	PG2	T1	1.18 ± 0.03 ^j^	0.34 ± 0.02 ^j^	1.05 ± 0.04 ^j^	1.95 ± 0.02 ^c^	0.53 ± 0.02 ^kl^
		T2	1.25 ± 0.03 ^i^	0.48 ± 0.04 ^g^	1.16 ± 0.03 ^hi^	1.88 ± 0.03 ^d^	0.61 ± 0.02 ^ij^
		T3	1.71 ± 0.03 ^e^	0.65 ± 0.02 ^c^	1.60 ± 0.04 ^c^	1.20 ± 0.02 ^k^	1.33 ± 0.04 ^c^
		T4	1.41 ± 0.03 ^g^	0.52 ± 0.03 ^f^	1.36 ± 0.03 ^e^	1.59 ± 0.04 ^h^	0.85 ± 0.03 ^f^
	PG3	T1	1.33 ± 0.03 ^h^	0.43 ± 0.02 ^h^	1.07 ± 0.02 ^j^	1.83 ± 0.03 ^e^	0.58 ± 0.01 ^jk^
		T2	1.35 ± 0.02 ^h^	0.54 ± 0.02 ^f^	1.19 ± 0.02 ^h^	1.68 ± 0.05 ^g^	0.70 ± 0.01 ^gh^
		T3	1.76 ± 0.03 ^d^	0.68 ± 0.02 ^b^	1.87 ± 0.02 ^b^	1.05 ± 0.04 ^l^	1.78 ± 0.09 ^b^
		T4	1.54 ± 0.03 ^f^	0.58 ± 0.01 ^e^	1.36 ± 0.03 ^e^	1.49 ± 0.02 ^i^	0.91 ± 0.01 ^ef^
	PG4	T1	1.67 ± 0.03 ^e^	0.49 ± 0.02 ^g^	1.13 ± 0.03 ^i^	1.73 ± 0.04 ^f^	0.65 ± 0.03 ^hi^
		T2	1.92 ± 0.03 ^c^	0.62 ± 0.02 ^d^	1.30 ± 0.03 ^f^	1.75 ± 0.02 ^f^	0.74 ± 0.02 ^g^
		T3	2.11 ± 0.04 ^a^	0.76 ± 0.03 ^a^	1.97 ± 0.02 ^a^	0.85 ± 0.03 ^m^	2.31 ± 0.06 ^a^
		T4	2.04 ± 0.05 ^b^	0.67 ± 0.02 ^bc^	1.45 ± 0.04 ^d^	1.39 ± 0.03 ^j^	1.04 ± 0.04 ^d^
**2020**	PG1	T1	1.05 ± 0.02 ^k^	0.28 ± 0.03 ^l^	1.01 ± 0.03 ^k^	2.16 ± 0.02 ^a^	0.45 ± 0.01 ^l^
		T2	1.07 ± 0.02 ^k^	0.30 ± 0.02 ^l^	1.04 ± 0.03 ^l^	2.01 ± 0.07 ^b^	0.50 ± 0.02 ^kl^
		T3	1.40 ± 0.02 ^h^	0.40 ± 0.02 ^j^	1.36 ± 0.01 ^l^	1.39 ± 0.02 ^i^	0.97 ± 0.00 ^e^
		T4	1.20 ± 0.02 ^j^	0.36 ± 0.03 ^k^	1.32 ± 0.02 ^efg^	1.62 ± 0.02 ^g^	0.81 ± 0.03 ^g^
	PG2	T1	1.22 ± 0.02 ^j^	0.39 ± 0.01 ^jk^	1.06 ± 0.03 ^g^	1.91 ± 0.03 ^c^	0.55 ± 0.03 ^k^
		T2	1.30 ± 0.03 ^i^	0.49 ± 0.01 ^h^	1.19 ± 0.05 ^kl^	1.81 ± 0.02 ^d^	0.65 ± 0.01 ^ij^
		T3	1.75 ± 0.02 ^d^	0.69 ± 0.02 ^c^	1.64 ± 0.03 ^hi^	1.18 ± 0.02 ^k^	1.38 ± 0.08 ^c^
		T4	1.49 ± 0.06 ^g^	0.57 ± 0.02 ^f^	1.40 ± 0.08 ^e^	1.53 ± 0.02 ^h^	0.91 ± 0.02 ^f^
	PG3	T1	1.31 ± 0.05 ^i^	0.46 ± 0.02 ^i^	1.10 ± 0.01 ^jk^	1.79 ± 0.03 ^d^	0.61 ± 0.01 ^j^
		T2	1.39 ± 0.01 ^h^	0.56 ± 0.03 ^f^	1.23 ± 0.04 ^h^	1.65 ± 0.06 ^fg^	0.74 ± 0.02 ^h^
		T3	1.81 ± 0.03 ^c^	0.72 ± 0.02 ^b^	1.91 ± 0.03 ^b^	1.00 ± 0.02 ^l^	1.91 ± 0.04 ^b^
		T4	1.63 ± 0.02 ^f^	0.62 ± 0.02 ^e^	1.39 ± 0.02 ^ef^	1.43 ± 0.01 ^i^	0.97 ± 0.01 ^e^
	PG4	T1	1.69 ± 0.03 ^e^	0.53 ± 0.02 ^g^	1.16 ± 0.02 ^ij^	1.68 ± 0.02 ^ef^	0.69 ± 0.00 ^i^
		T2	2.03 ± 0.05 ^b^	0.66 ± 0.01 ^d^	1.34 ± 0.03 ^fg^	1.70 ± 0.02 ^e^	0.78 ± 0.01 ^gh^
		T3	2.19 ± 0.03 ^a^	0.80 ± 0.03 ^a^	1.99 ± 0.02 ^a^	0.80 ± 0.01 ^m^	2.48 ± 0.02 ^a^
		T4	2.15 ± 0.04 ^a^	0.69 ± 0.03 ^c^	1.48 ± 0.04 ^d^	1.35 ± 0.03 ^j^	1.09 ± 0.02 ^d^
**F-test**	Phosphogypsum rates (PG)		***	***	***	***	***
	Inoculation (I)		***	***	***	***	***
	Interaction (PG × I)		***	***	***	***	***

Means of the same growing season designated with different letters indicate significant differences among treatments according to the Duncan test (*p* < 0.05). Values are the means ± standard deviation (SD) from 3 replicates (means ± SD). PG1—0 t ha^−1^; PG2—3 t ha^−1^; PG3—6 t ha^−1^; PG4—9 t ha^−1^; T1—*A. lipoferum* (control); T2—*A. lipoferum* + *B. coagulans*; T3—*A. lipoferum* + *B. circulance*, and T4—*A. lipoferum* + *B. subtilis*; ***—High significant.

**Table 4:** The combined effect of different rates of phosphogypsum and microbial inoculations on micro-nutrients (mg Kg^−1^) in maize leaves grown in salt-affected soils during the 2019 and 2020 seasons.

Year	Phosphogypsum Rates (PG)	Inoculation (I)	Zn	Mn	Fe	Cu
**2019**	PG1	T1	23.73 ± 0.32 ^m^	23.55 ± 0.38 ^n^	55.04 ± 0.22 ^j^	7.17 ± 0.06 ^h^
		T2	25.36 ± 0.45 ^l^	24.43 ± 0.27 ^m^	61.75 ± 0.11 ^i^	7.44 ± 0.04 ^g^
		T3	35.67 ± 0.32 ^h^	26.42 ± 0.32 ^k^	75.31 ± 0.19 ^efg^	7.74 ± 0.05 ^e^
		T4	28.82 ± 0.20 ^k^	25.19 ± 0.17 ^l^	66.29 ± 0.10 ^h^	7.60 ± 0.09 ^f^
	PG2	T1	29.84 ± 0.44 ^j^	27.45 ± 0.33 ^j^	67.69 ± 0.17 ^h^	7.59 ± 0.04 ^f^
		T2	32.79 ± 0.41 ^i^	29.95 ± 0.11 ^i^	73.04 ± 0.15 ^fg^	7.72 ± 0.03 ^e^
		T3	47.56 ± 0.42 ^c^	32.24 ± 0.29 ^g^	86.13 ± 0.12 ^c^	8.18 ± 0.05 ^c^
		T4	39.88 ± 0.40 ^f^	31.38 ± 0.14 ^h^	75.77 ± 6.80 ^ef^	7.91 ± 0.04 ^d^
	PG3	T1	37.69 ± 0.45 ^g^	31.34 ± 0.14 ^h^	72.40 ± 0.08 ^g^	7.77 ± 0.07 ^e^
		T2	41.37 ± 0.22 ^e^	33.79 ± 0.07 ^f^	77.68 ± 0.19 ^e^	7.96 ± 0.07 ^d^
		T3	51.55 ± 0.50 ^b^	37.56 ± 0.28 ^c^	91.13 ± 0.14 ^b^	8.26 ± 0.03 ^b^
		T4	46.99 ± 0.11 ^cd^	35.21 ± 0.06 ^e^	84.09 ± 0.08 ^cd^	8.12 ± 0.04 ^c^
	PG4	T1	40.13 ± 0.33 ^f^	33.71 ± 0.05 ^f^	81.09 ± 0.10 ^d^	7.92 ± 0.03 ^d^
		T2	46.69 ± 0.47 ^d^	36.49 ± 0.06 ^d^	85.36 ± 0.34 ^c^	8.16 ± 0.05 ^c^
		T3	59.38 ± 0.15 ^a^	40.48 ± 0.04 ^a^	94.24 ± 0.46 ^a^	8.48 ± 0.04 ^a^
		T4	51.33 ± 0.33 ^b^	38.65 ± 0.42 ^b^	90.52 ± 0.17 ^b^	8.28 ± 0.05 ^b^
**2020**	PG1	T1	24.21 ± 0.02 ^l^	23.74 ± 0.37 ^n^	55.45 ± 0.38 ^h^	7.25 ± 0.07 ^i^
		T2	25.80 ± 0.13 ^k^	24.65 ± 0.25 ^m^	61.90 ± 0.09 ^gh^	7.80 ± 0.12 ^g^
		T3	36.32 ± 0.37 ^g^	26.82 ± 0.25 ^k^	75.67 ± 0.26 ^de^	7.90 ± 0.03 ^f^
		T4	29.33 ± 0.60 ^j^	25.41 ± 0.32 ^l^	66.55 ± 0.29 ^fg^	7.76 ± 0.08 ^gh^
	PG2	T1	30.59 ± 0.45 ^i^	27.87 ± 0.05 ^j^	67.99 ± 0.10 ^efg^	7.70 ± 0.04 ^h^
		T2	33.18 ± 0.19 ^h^	30.69 ± 0.45 ^i^	61.16 ± 20.67 ^gh^	7.88 ± 0.04 ^f^
		T3	48.56 ± 0.49 ^c^	32.40 ± 0.33 ^g^	86.43 ± 0.31 ^abc^	8.26 ± 0.04 ^d^
		T4	40.84 ± 0.18 ^e^	31.54 ± 0.13 ^h^	79.13 ± 1.75 ^cd^	8.05 ± 0.07 ^e^
	PG3	T1	38.77 ± 0.38 ^f^	31.65 ± 0.21 ^h^	72.70 ± 0.28 ^def^	7.87 ± 0.07 ^f^
		T2	42.24 ± 0.55 ^d^	34.03 ± 0.08 ^f^	77.97 ± 0.07 ^cd^	8.06 ± 0.06 ^e^
		T3	52.12 ± 0.23 ^b^	37.76 ± 0.17 ^c^	91.95 ± 0.08 ^ab^	8.34 ± 0.04 ^c^
		T4	47.77 ± 0.67 ^c^	35.62 ± 0.39 ^e^	84.55 ± 0.29 ^bc^	8.24 ± 0.05 ^d^
	PG4	T1	40.58 ± 0.43 ^e^	33.92 ± 0.07 ^f^	81.36 ± 0.27 ^cd^	8.06 ± 0.06 ^e^
		T2	47.90 ± 1.65 ^c^	36.82 ± 0.23 ^d^	85.65 ± 0.33 ^abc^	8.25 ± 0.05 ^d^
		T3	60.14 ± 0.18 ^a^	41.39 ± 0.34 ^a^	94.40 ± 0.36 ^a^	8.58 ± 0.02 ^a^
		T4	52.00 ± 0.13 ^b^	38.79 ± 0.44 ^b^	90.91 ± 0.12 ^ab^	8.43 ± 0.05 ^b^
**F-test**	Phosphogypsum rates (PG)		***	***	***	***
	Inoculation (I)		***	***	***	***
	Interaction (PG × I)		***	***	***	***

Means of the same growing season designated with different letters indicate significant differences among treatments according to the Duncan test (*p* < 0.05). Values are the means ± standard deviation (SD) from 3 replicates (means ± SD). PG1—0 t ha^−1^; PG2—3 t ha^−1^; PG3—6 t ha^−1^; PG4—9 t ha^−1^; T1—*A. lipoferum* (control); T2—*A. lipoferum* + *B. coagulans*; T3—*A. lipoferum* + *B. circulance*, and T4—*A. lipoferum* + *B. subtilis*; ***—High significant.

**Table 5:** The combined effect of different rates of phosphogypsum and microbial inoculations on yield and yield component of maize plants grown in salt-affected soils during the 2019 and 2020 seasons.

Year	Phosphogypsum Rates (PG)	Inoculation (I)	Ear Length (cm)	Ear Diameter (cm)	Grains/Ear	100-Grain Weight (g)	Grain Yield (kg ha^−1^)
**2019**	PG1	T1	15.97 ± 0.45 ^j^	3.53 ± 0.12 ^j^	387.33 ± 4.73 ^k^	31.70 ± 0.10 ^l^	4746.66 ± 45.09 ^n^
		T2	17.17 ± 0.21 ^i^	3.70 ± 0.10 i	405.00 ± 3.61 ^j^	32.16 ± 0.21 ^k^	5117.66 ± 14.01 ^k^
		T3	20.20 ± 0.26 ^d^	3.90 ± 0.10 ^h^	425.00 ± 3.61 ^h^	36.20 ± 0.70 ^c^	5729.66 ± 28.50 ^e^
		T4	19.17 ± 0.15 ^e^	4.13 ± 0.06 ^fg^	428.66 ± 1.53 ^g^	33.50 ± 0.40 ^fg^	5443.00 ± 42.14 ^h^
	PG2	T1	17.33 ± 0.42 ^hi^	4.00 ± 0.10 ^gh^	422.00 ± 3.00 ^i^	32.36 ± 0.15 ^jk^	5006.33 ± 16.26 ^l^
		T2	18.87 ± 0.15 ^e^	4.16 ± 0.06 ^f^	429.33 ± 1.53 ^g^	32.70 ± 0.10 ^ij^	5359.00 ± 51.12 ^i^
		T3	22.63 ± 0.15 ^b^	4.56 ± 0.06 ^abc^	449.66 ± 1.15 ^b^	37.66 ± 0.21 ^b^	5936.66 ± 20.82 ^c^
		T4	20.03 ± 0.21 ^d^	4.43 ± 0.06 ^cde^	437.66 ± 1.53 ^de^	33.86 ± 0.15 ^f^	5619.66 ± 17.56 ^f^
	PG3	T1	17.67 ± 0.21 ^gh^	4.10 ± 0.10 ^fg^	426.66 ± 3.21 ^gh^	32.60 ± 0.10 ^ij^	4910.00 ± 30.00 ^m^
		T2	17.90 ± 0.10 ^fg^	4.36 ± 0.06 ^de^	435.33 ± 2.08 ^ef^	33.13 ± 0.21 ^gh^	5431.66 ± 33.08 ^h^
		T3	22.87 ± 0.06 ^b^	4.63 ± 0.12 ^ab^	452.00 ± 1.00 ^b^	37.93 ± 0.25 ^b^	6028.66 ± 23.07 ^b^
		T4	20.20 ± 0.26 ^d^	4.56 ± 0.06 ^abc^	439.00 ± 2.00 ^d^	34.53 ± 0.58 ^e^	5752.33 ± 45.35 ^e^
	PG4	T1	18.23 ± 0.21 ^f^	4.33 ± 0.06 ^e^	433.33 ± 3.51 ^f^	32.96 ± 0.12 ^hi^	5236.33 ± 25.11 ^j^
		T2	19.17 ± 0.21 ^e^	4.50 ± 0.10 ^bcd^	436.66 ± 2.31 ^de^	33.50 ± 0.26 ^fg^	5534.66 ± 26.76 ^g^
		T3	24.20 ± 0.26 ^a^	4.70 ± 0.10 ^a^	458.00 ± 1.00 ^a^	38.46 ± 0.15 ^a^	6235.33 ± 20.43 ^a^
		T4	22.10 ± 0.10 ^c^	4.50 ± 0.10 ^bcd^	445.00 ± 1.00 ^c^	35.70 ± 0.10 ^d^	5846.33 ± 21.22 ^d^
**2020**	PG1	T1	16.13 ± 0.40 ^j^	3.60 ± 0.10 ^g^	389.00 ± 3.61 ^j^	31.83 ± 0.21 ^h^	4805.67 ± 45.09 ^n^
		T2	17.27 ± 0.21 ^i^	4.03 ± 0.61 ^f^	414.00 ± 12.49 ^i^	32.33 ± 0.06 ^gh^	5186.67 ± 14.01 ^k^
		T3	20.10 ± 0.26 ^d^	4.00 ± 0.10 ^f^	427.00 ± 4.36 ^gh^	35.00 ± 2.00 ^bc^	5802.00 ± 31.05 ^e^
		T4	19.30 ± 0.10 ^e^	4.20 ± 0.10 ^def^	431.00 ± 1.73 ^fg^	33.57 ± 0.35 ^ef^	5515.00 ± 40.73 ^h^
	PG2	T1	17.37 ± 0.38 ^i^	3.97 ± 0.06 ^f^	422.33 ± 4.51 ^h^	32.37 ± 0.06 ^gh^	5065.33 ± 16.26 ^l^
		T2	18.90 ± 0.20 ^f^	4.23 ± 0.06 ^c–f^	430.67 ± 2.08 ^fg^	32.73 ± 0.06 ^fgh^	5428.00 ± 51.12 ^i^
		T3	22.63 ± 0.12 ^bc^	4.53 ± 0.15 ^abc^	453.33 ± 2.89 ^b^	37.80 ± 0.20 ^a^	6010.67 ± 20.82 ^c^
		T4	20.23 ± 0.21 ^d^	4.47 ± 0.06 ^a–d^	439.67 ± 2.08 ^de^	33.97 ± 0.15 ^de^	5690.67 ± 17.56 ^f^
	PG3	T1	17.77 ± 0.12 ^h^	4.10 ± 0.10 ^ef^	427.33 ± 3.06 ^gh^	32.63 ± 0.15 ^gh^	4969.00 ± 30.00 ^m^
		T2	18.00 ± 0.17 ^gh^	4.40 ± 0.10 ^b–e^	437.67 ± 1.15 ^de^	33.23 ± 0.29 ^efg^	5500.67 ± 33.08 ^h^
		T3	23.00 ± 0.10 ^b^	4.73 ± 0.12 ^a^	450.67 ± 5.51 ^b^	38.30 ± 0.20 ^a^	6102.67 ± 23.07 ^b^
		T4	20.47 ± 0.21 ^d^	4.57 ± 0.06 ^ab^	442.00 ± 2.00 ^cd^	34.50 ± 0.44 ^cd^	5823.33 ± 45.35 ^e^
	PG4	T1	18.30 ± 0.20 ^g^	4.23 ± 0.15 ^c–f^	434.33 ± 3.06 ^ef^	33.10 ± 0.26 ^efg^	5295.33 ± 25.11 ^j^
		T2	19.13 ± 0.42 ^ef^	4.53 ± 0.06 ^abc^	438.67 ± 3.21 ^de^	33.63 ± 0.21 ^def^	5603.67 ± 26.76 ^g^
		T3	24.27 ± 0.31 ^a^	4.73 ± 0.06 ^a^	460.00 ± 1.00 ^a^	38.70 ± 0.26 ^a^	6309.33 ± 20.43 ^a^
		T4	22.50 ± 0.20 ^c^	4.67 ± 0.06 ^ab^	447.33 ± 2.08 ^bc^	35.87 ± 0.12 ^b^	5917.33 ± 21.22 ^d^
**F-test**	Phosphogypsum rates (PG)		***	***	***	***	***
	Inoculation (I)		***	***	***	***	***
	Interaction (PG × I)		***	***	***	***	***

Means of the same growing season designated with different letters indicate significant differences among treatments according to the Duncan test (*p* < 0.05). Values are the means ± standard deviation (SD) from 3 replicates (means ± SD). PG1—0 t ha^−1^; PG2—3 t ha^−1^; PG3—6 t ha^−1^; PG4—9 t ha^−1^; T1—*A. lipoferum* (control); T2—*A. lipoferum* + *B. coagulans*; T3—*A. lipoferum* + *B. circulance*, and T4—*A. lipoferum* + *B. subtilis*; ***—High significant.

**Table 6:** The physical, chemical, and biological characteristics of the experimental area during the two growing seasons of 2019 and 2020.

Season	Character			
	pH (1:2.5)	EC (dS m^−^)	O.M (%)	ESP (%)
**2019**	8.22 ± 0.02	7.33 ± 0.04	1.56 ± 0.01	21.27 ± 0.17
**2020**	8.18 ± 0.04	7.41 ± 0.03	1.65 ± 0.04	20. 72 ± 0.32
**Season**	**Particle Size Distribution (%)**			
	**Sand**	**Silt**	**Clay**	**Texture grade**
**2019**	28.22 ± 1.66	24.11 ± 1.83	47.67 ± 2.01	Clayey
**2020**	28.76 ± 1.78	24.60 ± 1.92	46.64 ± 2.11	Clayey
**Season**	**Soluble Cations (meq L** **^−1^)**			
	**Ca^2+^**	**Mg^2+^**	**Na^+^**	**K^+^**
**2019**	7.32 ± 0.66	5.12 ± 1.22	24.22 ± 2.09	0.38 ± 0.09
**2020**	8.11 ± 0.51	5.38 ± 1.38	23.67 ± 2.29	0.34 ± 0.05
**Season**	**Soluble Anions (meq L^−1^)**			
	**CO_3_^2^^−^**	**HCO_3_^−^**	**Cl^−^**	**SO_4_^2^^−^**
**2019**	-	4.01 ± 0.55	20.00 ± 1.29	13.03 ± 2.11
**2020**	-	3.98 ± 0.37	21.13 ± 1.86	12.39 ± 2.02
**Season**	**Available Macronutrients (mg kg** **^−1^)**			
	**N**	**P**		**K**
**2019**	8.81 ± 0.71	7.12 ± 1.12		322 ± 17.23
**2020**	9.29 ± 0.45	7.96 ±1.32		341 ± 14.11
**Season**	**Total Counts of Microbes (CFU × 10^5^ g** **^−1^ dry soil)**			
	*Bacillus* *		***Azospirillum***	
**2019**	44 ± 2.12		23 ± 1.25	
**2020**	58 ± 2.65		35 ± 1.44	

±: Standard deviation; EC: electrical conductivity (measured in soil paste extract); O.M: organic matter (measured by modified Walkly and Black method, [56]); ESP: exchangeable sodium percentage; *—Total counts of *Bacillus* were done by pasteurization in a water bath at 70 °C for 10 min to kill the vegetative cells.

**Table 7:** Meteorological data for the two summer growing seasons of 2019 and 2020.

Season	2019				
Month	Temperature (°C)		Wind Speed (km day^−1^)	RH (%)	Rainfall (mm month^−1^)
	Max	Min			
**May**	33.7	16.2	122.3	70.1	0.0
**June**	35.4	18.4	117.5	66.3	0.0
**July**	36.5	22.5	101.2	65.7	0.0
**August**	36.7	19.2	94.2	63.2	0.0
**September**	32.3	18.2	84.7	67.8	0.0
**Season**	**2020**				
**Month**	**Temperature (°C)**		**Wind Speed** **(km day^−1^)**	**RH (%)**	**Rainfall** **(mm month^−1^)**
	**Max**	**Min**			
**May**	33.1	17.2	125.3	68.5	0.0
**June**	36.2	16.4	115.2	65.7	0.0
**July**	35.4	22.3	105.8	64.2	0.0
**August**	38.6	23.1	93.2	62.0	0.0
**September**	37.1	22.5	85.2	45.9	0.0

Max—maximum, min—minimum, RH—relative humidity.

## Data Availability

The data that supports the findings of this study are contained within the article or supplementary material and available from the corresponding author upon reasonable request.
